# Search for Venous Endothelial Biomarkers Heralding Venous Thromboembolism in Space: A Qualitative Systematic Review of Terrestrial Studies

**DOI:** 10.3389/fphys.2022.885183

**Published:** 2022-04-27

**Authors:** Katie Harris, Jonathan Michael Laws, Antoine Elias, David Andrew Green, Nandu Goswami, Jens Jordan, Tovy Haber Kamine, Lucia Mazzolai, Lonnie G. Petersen, Andrew James Winnard, Tobias Weber

**Affiliations:** ^1^ Faculty of Medicine, Memorial University of Newfoundland, St. John’s, NL, Canada; ^2^ Space Biomedicine Systematic Review Methods Group, Wylam, United Kingdom; ^3^ Department of Vascular Medicine, Sainte Musse Hospital, Toulon La Seyne Hospital Centre, Toulon, France; ^4^ European Astronaut Centre (EAC), European Space Agency, Space Medicine Team (HRE-OM), Cologne, Germany; ^5^ KBR GmbH, Cologne, Germany; ^6^ King’s College London, Centre of Human & Applied Physiological Sciences, London, United Kingdom; ^7^ Division of Physiology, Otto Löwi Research Center for Vascular Biology, Immunity and Inflammation, Medical University of Graz, Graz, Austria; ^8^ Mohammed Bin Rashid University of Medicine and Applied Health Sciences, Dubai, United Arab Emirates; ^9^ Institute of Aerospace Medicine, German Aerospace Center, Cologne, Germany; ^10^ Division of Trauma, Acute Care Surgery, and Surgical Critical Care, Baystate Medical Center, Springfield, MA, United States; ^11^ Department of Angiology, Centre Hospitalier Universitaire Vaudois, Lausanne, Switzerland; ^12^ Mechanical and Aerospace Engineering, University of California, San Diego, San Diego, CA, United States; ^13^ Department of Radiology, University of California, San Diego, San Diego, CA, United States

**Keywords:** venous thrombosis, biomarker, Virchow’s triad, venous thromboembolism, VTE, DVT, Endothelial (dys)function, spaceflight

## Abstract

**Background:** The recent discovery of a venous thrombosis in the internal jugular vein of an astronaut has highlighted the need to predict the risk of venous thromboembolism in otherwise healthy individuals (VTE) in space. Virchow’s triad defines the three classic risk factors for VTE: blood stasis, hypercoagulability, and endothelial disruption/dysfunction. Among these risk factors, venous endothelial disruption/dysfunction remains incompletely understood, making it difficult to accurately predict risk, set up relevant prophylactic measures and initiate timely treatment of VTE, especially in an extreme environment.

**Methods:** A qualitative systematic review focused on endothelial disruption/dysfunction was conducted following the guidelines produced by the Space Biomedicine Systematic Review Group, which are based on Cochrane review guidelines. We aimed to assess the venous endothelial biochemical and imaging markers that may predict increased risk of VTE during spaceflight by surveying the existing knowledge base surrounding these markers in analogous populations to astronauts on the ground.

**Results:** Limited imaging markers related to endothelial dysfunction that were outside the bounds of routine clinical practice were identified. While multiple potential biomarkers were identified that may provide insight into the etiology of endothelial dysfunction and its link to future VTE, insufficient prospective evidence is available to formally recommend screening potential astronauts or healthy patients with any currently available novel biomarker.

**Conclusion:** Our review highlights a critical knowledge gap regarding the role biomarkers of venous endothelial disruption have in predicting and identifying VTE. Future population-based prospective studies are required to link potential risk factors and biomarkers for venous endothelial dysfunction to occurrence of VTE.

## Introduction

The recent incidental discovery of an internal jugular vein thrombosis in an astronaut during long duration spaceflight on the International Space Station (Auñón-Chancellor et al., 2020) has brought predicting the risk of VTE in otherwise healthy individuals to the forefront of the space medicine research community’s interests. While the VTE in question was treated successfully in space with enoxaparin (Auñón-Chancellor et al., 2020), a similar situation during a mission beyond low Earth orbit, far from ground-based medical intervention, could result in significant mission disruption or possible loss of crew life. Therefore, predicting the baseline VTE risk of any given astronaut candidate during medical screening has high value as a tool for prevention of VTE in space. While known thrombophilia disorders are already screened for and are cause for medical exclusion, the complex etiology of VTE touts the potential for novel biomarkers to be used to assess risk. Furthermore, identifying biomarkers that could indicate the precursor processes of VTE in early stages onboard future diagnostic assays in space could be used to justify prophylactic measures such as low dose anticoagulants.

Virchow’s triad outlines the classic risk factors for clot formation: blood stasis, hypercoagulability, and endothelial disruption/dysfunction (Vazquez-Garza et al., 2017). Immobilizations such as bed rest, pregnancy, and plaster casts, all associated with blood flow stasis, are among known common risk factors for venous thromboembolism (VTE) (Reitsma et al., 2012), and spaceflight arguably constitutes another (Kim et al., 2021). Established risks such as malignancies, chemotherapy, oral contraceptives, hormone replacement therapy, obesity, infection, inflammatory disease, lupus, and genetic factors predispose to VTE at least in part through hypercoagulability due to increased clotting factor concentrations or reduced fibrinolytic factor concentrations and innate immunity. Others, such as venous catheters, trauma, or surgery may primarily affect endothelial integrity (Reitsma et al., 2012; Gordon and Lombard, 2017). The mechanisms mediating spaceflight-associated VTE are still elusive, however, all components of Virchow’s triad have been implicated (Limper et al., 2021).

Among the components of Virchow’s triad, which collectively determine VTE risk, endothelial disruption/dysfunction of the venous endothelium received the least attention (Yau et al., 2015). The arterial endothelial function has been studied in great detail due to the association with atherosclerosis and cardiac events (Tsai et al., 2002) much less is known about the venous endothelium. While atherosclerosis has also been linked with occurrence of VTE (Poredos and Jezovnik, 2011), not all predictors for atherosclerotic disease cross over to VTE. Therefore, the etiological differences between the two systems, such as lower shear stress and differing clot composition in the venous system, must be considered (Tsai et al., 2002; Vazquez-Garza et al., 2017).

**FIGURE 1:**
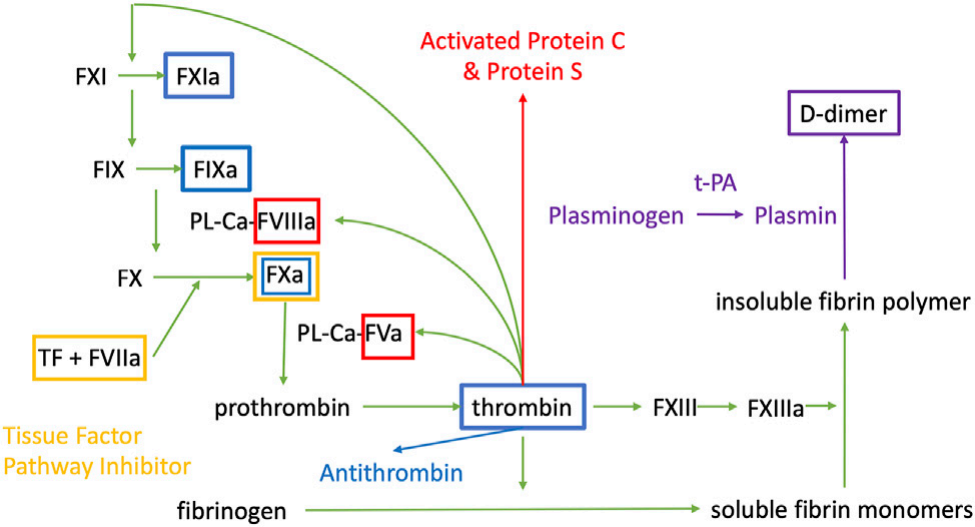
Coagulation cascade. PL are phospholipids, Ca is Calcium, F is for Factor, a indicates that it is the activated form of the factor. t-PA is tissue plasminogen activator. The colour of the boxes correspond to the substance that is inhibiting the factor contained in the box.

The endothelium together with the smooth muscle cells controls blood flow and vascular tone, is targeted by pro-inflammatory cytokines and leukocytes, and provides a surface for the expression of molecules triggering the coagulation cascade (Yau et al., 2015). A schematic summary of the coagulation cascade is provided in Figure 1 to frame the discussion in this review.

Whenever the endothelium is damaged, the initial step is vasoconstriction to limit blood flow and therefore hemorrhage. The ruptured endothelial barrier exposes prothrombic subendothelial factors such as Von Willebrand Factor (vWF) and collagen to blood promoting platelet adhesion (Palta et al., 2014). In turn, activated coagulation factors form complexes on the platelets, promoting the formation of a cross linked fibrin clot (Palta et al., 2014).

Once the coagulation cascade is activated, the anticoagulant and fibrinolytic systems are also activated, giving rise to complex interactions. Upon thrombin activation, a negative feedback loop induces activation of natural anticoagulants Protein C (PC) and Protein S (PS). PC and PS regulate the activity of activated factor V (FVa) and activated factor VIII (FVIIIa) (Palta et al., 2014). The fibrinolytic system is also activated during coagulation and tissue factor plasminogen inhibitor (TFPI) inhibits activated factor X, tissue factor, and activated factor VII complex (Palta et al., 2014). Thrombin generates antithrombin, which inhibits thrombin and Factors IXa, Xa, and XIa, while endothelial cells produce prostaglandin and nitric oxide (NO) which inhibit vasoconstriction and platelet aggregation (Palta et al., 2014). Plasmin is formed from plasminogen *via* tissue plasminogen activator (tPA), which then lyses the insoluble fibrin polymer to D-dimer and fibrin degradation products. Thrombomodulin binds to thrombin to prevent clot formation on undamaged endothelium (Palta et al., 2014).

The multitude of factors and co-factors involved in the regulation of coagulation provide many possibilities for VTE risk assessment and diagnosis based on factor levels. While D-dimers (fibrin degradation products, increased following thrombus formation) are traditionally used in the algorithm of preclinical assessment of VTE suspicion because of their high negative predictive value, their lack of specificity does not allow their use for VTE diagnosis which necessitates imaging studies (Barnes et al., 2008; Jacobs et al., 2016). Barnes et al. (Barnes et al., 2008) conducted a narrative review focused on potential biomarkers for the specific diagnosis of VTE, such as P-selectin, E-selectin, microparticles, thrombin, fibrin monomers, and interleukin 10 (IL-10). They found that several studies determined that P-selectin was elevated in patients with deep venous thrombosis (DVT), which may decrease after the acute event (Barnes et al., 2008).

Microparticles have less evidence, but are also elevated in acute settings (Barnes et al., 2008). Genetic mutations associated with the E-selectin and IL-10 genes additionally predicted increased risk of DVT. Looking forward, the authors suggested that these biomarkers may be able to help predict recurrence of VTE and the severity of thrombosis (Barnes et al., 2008). A recent follow up review by Jacobs et al. (Jacobs et al., 2016) came to similar conclusion regarding P-selectin and D-dimer.

The purpose of the present systematic review is primarily to identify potential biological and imaging markers related to endothelial dysfunction or VTE events in terrestrial medicine that could be utilized to elucidate VTE mechanisms in space. Subsequently, these biomarkers could be systematically tested to determine their performance in identifying spaceflight-associated VTE risk early on. On potential application is risk prediction during space flight crew selection. Moreover, biomarker testing could be utilized to guide preventive measures in space, such as low dose anticoagulation, before overt VTE occurs. The present study seeks to synthesize themes linking markers for endothelial disruption and/or dysfunction with the occurrence of VTE and generate recommendations for future research.

## Methods

### Search Strategy

A range of terms (Supplementary Table S1) were used in combinations to search PubMed, Web of Science, and Cochrane Reviews and Trials databases between June 24th-25th 2021. Initial pre-scoping was performed to determine appropriate search terms that would capture an adequate number of papers to reach knowledge saturation. In databases where Medical Subject Headings (MeSH) terms cannot be used, the same terms were searched without the MeSH identifier. The European Space Agency (ESA) Topical Team on VTE contributed with their expertise to ensure all appropriate keywords were included. The qualitative systematic review analysis method was chosen due to high heterogeneity of available evidence, and the lack of consistent study protocols and outcomes that would allow metanalysis of previously published findings. The population of interest was healthy individuals with no prior risk factors for VTE, either as main study subjects or controls. The Interest was linking VTE with the venous endothelial system. No limitation on controls were specified. The Outcomes included structural biomarkers, venous mechanical properties, venous flow properties, symptoms, circulating biomarkers, endothelial markers, blood cell counts, thrombelastometry, platelet aggregation, coagulation times, thrombin generation, fibrinolytic values/endothelial activation, and pro- and anti-coagulatory factors. The full Population, Interest, Control, and Outcome (PICO) table used to define the present search criteria, along with the search strategy implemented can be found in the Supplementary Material as Table 1.

**TABLE 1:** Risk of Bias assessment.

Paper	Study Design	Participant Selection Risk	Confounding Variables Risk	Measurement of Exposure Risk	Blinding of Outcome Assessments Risk	Incomplete Outcome Data Risk	Selective Outcome Reporting Risk
Alvarado-Moreno et al. (2016)	Case-Control	Low	Low	Low	Low	Low	Low
Ansari et al. (2006)	Before-after	Low	Low	Low	Low	Low	Low
Bombeli et al. (2002)	Case-Control	Low	Low	Low	Low	Low	Low
Bucek et al. (2003)	Before-after	Low	Low	Low	Low	Low	Low
Chirinos et al. (2005)	Case-Control	Low	Low	Low	Low	Low	Low
Falkon et al. (1992)	Case-Control	Low	High	Low	Low	Low	Low
Grimaudo et al. (1992)	Case-Control	Unclear	Unclear	Low	Low	Low	Low
Jezovnik et al. (2010)	Case-Control	Low	High	Low	Low	Low	Low
Martos et al. (2020)	Case-Control	Low	Low	Low	Low	Low	Low
Masoud et al. (2008)	Before-after	Low	Low	Low	Low	Low	Low
Meltzer et al. (2010)	Case-Control	Low	Low	Low	Low	Low	Low
Poredos et al. (2011)	Case-Control	Low	High	Low	Low	Low	Low
Ruhl et al. (2014)	Case-Control	Low	Unclear	Low	Low	Low	Low
Sidelmann et al. (2008)	Case-Control	Unclear	Unclear	Low	Low	Low	Low
Torres et al. (2017)	Case-Control	High	High	Low	Low	Low	Low
van Aken et al. (2000)	Case-Control	Low	Unclear	Low	Low	Low	Low
Zapponi et al. (2014)	Case-Control	Low	Unclear	Low	Low	Unclear	Unclear

### Inclusion Criteria

**FIGURE 2:**
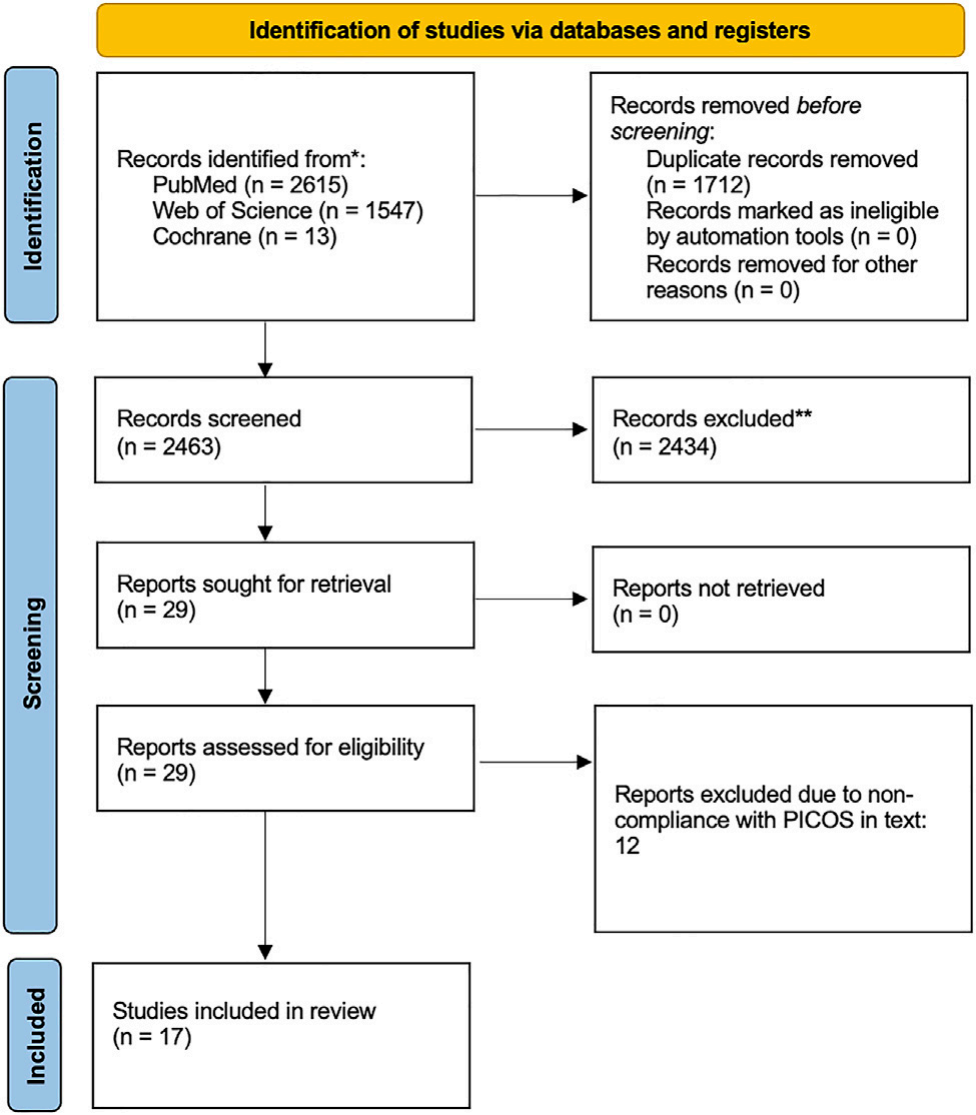
Paper Screening Flow. **Papers excluded due to lack of adherence to PICOS.

Any studies that did not meet the inclusion criteria were excluded. Included papers were original studies that were available as full texts and published in English. No limitation on publication period was used. The PRISMA standards (Preferred Reporting Items for Systematic reviews and Meta Analyses) checklist moher et al. was followed to ensure gold standard, transparent, and complete reporting of results. The PRISMA flow diagram is shown in Figure 2.

### Study Selection and Data Extraction

The initial screening of documents, using abstracts and titles, was carried out using the Rayyan systematic review online application (Ouzzani et al., 2016). Two authors screened the papers for adherence to the search criteria together (unblinded), using a third author as a tie breaker for any papers which they could not agree upon. Included papers were original studies on healthy humans (either as the main population or control group) that focused on linking endothelial biomarkers to the venous system in terms of VTE risk. Common reasons for papers to be excluded were that they were only performed *in vitro* or in cell models, that the study population lacked healthy controls or healthy subjects (ie. no known risk factors for VTE), or that the papers were reviews. Twelve papers were further excluded upon reading the full texts. If it was unclear from the initial screening whether a study met the inclusion criteria, the full text of the document was obtained. NVivo12 (QSR International Pty Ltd. (2018) NVivo (Version 12), https://www.qsrinternational.com/nvivo-qualitative-data-analysis-software/home) was used to extract data from each manuscript.

### Quality Assessment

The quality of included studies was assessed using the Risk of Bias Assessment Tool for Nonrandomized Studies RoBANS (Kim et al., 2013). RoBANS assesses the quality of non-randomized literature across six domains: the selection of participants; confounding variables; measurement of exposure; blinding of outcome assessments; incomplete outcome data; and selective outcome reporting, with each study ranking either low, unclear or high risk (Kim et al., 2013). The ranking of the included papers is shown above in Table 1.

### Data Analysis

Thematic analysis (Braun and Clarke, 2006; Braun et al., 2019) and thematic synthesis (Thomas and Harden, 2008) following the qualitative systematic review methods guide by Winnard et al. (2021) were used. Thematic analysis is split into 6 stages: The first stage involved reading all the included papers to identify relevant concepts and themes. The second stage was initial coding of included papers using NVivo12 (QSR International Pty Ltd. (2018) NVivo (Version 12), https://www.qsrinternational.com/nvivo-qualitative-data-analysis-software/home). The third was the grouping of codes in broader themes, the fourth reviewing the themes. The fifth step involved defining criteria for each theme, before the sixth and final step of creating a thematic analysis map.

## Results

The study population and sample collection time for each of the included studies is shown in Table 2. While all included papers looked at healthy populations either as the cohort or as the control group, the sample collection time for the participants affected by VTE in studies which had a case group varied widely, and was primarily after an acute VTE event, rather than before. This resulted in very little predictive value in the biomarkers studied. The two prospective studies, Ansari et al. (Ansari et al., 2006) and Masoud et al. (Masoud et al., 2008) did not result in any VTE occurrence with their relative interventions.

**TABLE 2:** Sample collection time relative to VTE event in selected papers.

Paper	Study Participants	Sample collection time for VTE population
Alvarado-Moreno et al. (2016)	Patients with recurrent VTE episodes and healthy controls	Last VTE episode was >6 months before sample collection
Ansari et al. (2006)	Healthy male volunteers exposed to prolonged seated immobility	Sample collected before and after 8 h of sitting, no VTE occurrence
Bombeli et al. (2002)	Patients with history of VTE and healthy controls	Last VTE episode was >3 months before sample collection
Bucek et al. (2003)	Patients with suspected DVT, confirmed with imaging after study	Sample collected before DVT confirmed, during symptomatic phase
Chirinos et al. (2005)	Patients with VTE and healthy controls	Sampled collected during symptomatic phase
Falkon et al. (1992)	Patients with history of VTE and healthy controls	Sample collected before and after 20 min of venous stasis
Grimaudo et al. (1992)	Patients with history of VTE and healthy controls	Sampled collected before and after 10 and 20 min of venous occlusion
Jezovnik et al. (2010)	Patients with first idiopathic VTE and healthy controls	Samples collected 2–4 months after first event
Martos et al. (2020)	Patients with VTE and healthy controls	Last VTE episode was >6 months before sample collection
Masoud et al. (2008)	Healthy volunteers	Samples collected at rest and before and after 15, 30, and 60 min of still standing, no VTE occurrence
Meltzer et al. (2010)	Patients with a history of VTE and healthy controls	Samples collected between 95–877 days after thrombosis
Poredos et al. (2011)	Patients with a history of VTE and healthy controls	Samples collected 2–4 months after initial presentation
Ruhl et al. (2014)	Thrombophilia patients and healthy controls	Samples collected 10 min after bed rest and after VOT.
Sidelmann et al. (2008)	Patients with DVT and controls with symptoms but no DVT.	Samples collected during the symptomatic phase
Torres et al. (2017)	Patients with VTE and healthy controls	Samples collected >1 month after initial presentation
van Aken et al. (2000)	Patients with recurrent VTE and healthy controls	Samples collected at least >1 ear since last episode
Zapponi et al. (2014)	Patients with VTE on oral anticoagulation and healthy controls	Samples collected after at least >6months of oral anticoagulation

**FIGURE 3:**
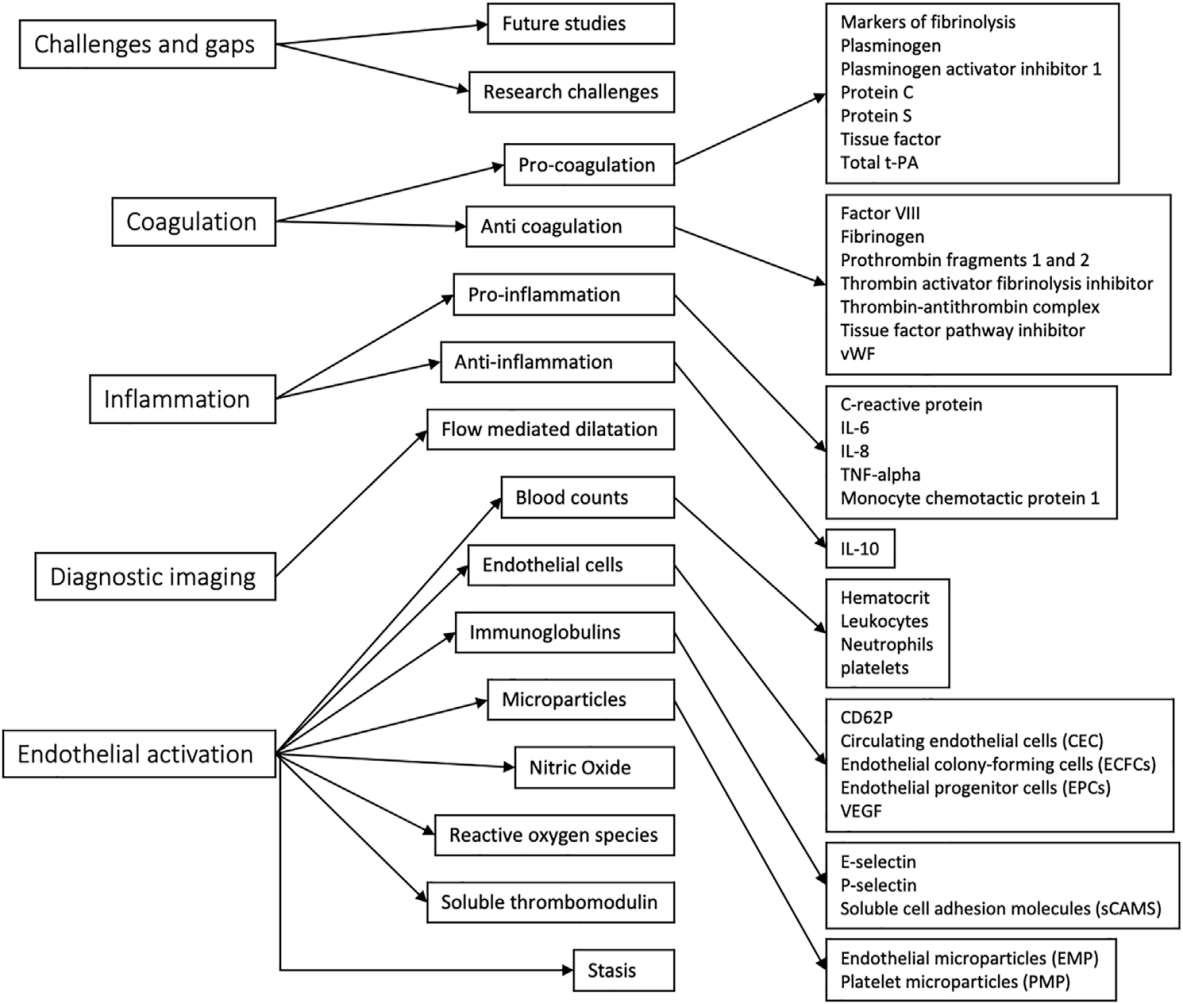
Thematic map, progressing from major themes on the left to minor themes to codes.

The thematic map generated through NVivo analysis is shown in Figure 3. Individual themes were first identified, then grouped into larger themes for aid analysis. While these themes loosely followed the PICOS distribution of search terms, coagulation, inflammation, and endothelial activation arose as the most appropriate umbrella themes for the key points of focus in the included papers. The criteria for inclusion of each code in the respective themes are explained in Table 3.

**TABLE 3:** Theme Criterion.

Major theme	Criterion
Challenges and gaps	Any acknowledged barrier to completing the research studies included, as well as future directions for research to proceed in were included in this theme
Coagulation	Pro- and anti-coagulation factors linked to the endothelium were included in this theme
Inflammation	Pro- and anti-inflammation factors linked to the endothelium were included in this theme, which was linked to the coagulation theme in terms of etiology of VTE.
Diagnostic Imaging	Imaging used to confirm suspicion of VTE or used in the studies to identify risk of VTE was included in this theme
Endothelial activation	Factors linked to activation of the endothelium in their production or proliferation were included in this theme. Indicators of endothelial activation were also included

## Discussion

In total, 17 papers that linked biomarkers of endothelial disruption to VTE and the venous endothelial system were included. Five broad themes were identified, which were coagulation, endothelial activation, inflammation, the role of diagnostic imaging, and challenges and gaps. All other identified themes were grouped under these categories for thematic analysis.

### Relevant Biomarkers for Coagulation, Endothelial Activation, and Inflammation

#### Coagulation and Endothelial Dysfunction in VTE Risk

The biomarkers related to coagulation are intuitively linked to the prediction of VTE risk. Therefore, it is not surprising that coagulation arose as an umbrella theme for this qualitative analysis. The tissue plasminogen activator/plasminogen activator inhibitor-1 (tPA/PAI-1) ratio is often considered a marker of ongoing fibrinolysis, similar to the D-dimer. Vascular endothelium promotes fibrinolysis due to release of tPA from vessel wall (Falkon et al., 1992), while PAI-1 is produced by endothelium and liver (Falkon et al., 1992). The linkage of the state of the vessel wall to the coagulation factors complicates the picture and makes it difficult to fully separate endothelial dysfunction/activation from coagulation factors, hence the discussion of these two themes together. For example, shear stress has been linked to increased levels of tPA and PAI-1 (Masoud et al., 2008) and enhanced expression of tissue factor (TF).

When measured at different sites (ie. arm and leg) in the same person undergoing venous stasis due to prolonged sitting, the changes in total tPA between the two sites did not correlate, which could be influenced by reduced haptic clearance of tPA or local endothelial activation effects due to localized venous stasis (Ansari et al., 2006), whereas the increases in PAI-1 were strongly correlated between body sites, indicating a more systemic physiological response. Another study found that when a local stimulus is applied to the endothelium through targeted venous stasis, there is only little correlation between tPA and PAI-1, indicating both endothelial and systemic forces working at different secretory levels (Falkon et al., 1992). Baseline values of tPA and plasmin-alpha2-antiplasmin (PAP) were slightly higher for thrombophilic patients than healthy probands in (Rühl et al., 2014), but this study found that the release of tPA from endothelial cells during short stasis was not influenced by presence of thrombophilic risk factors or clinically relevant arterial phenomenon.

One way to determine the contribution of endothelial factors to coagulation is to directly stimulate the endothelium. Studies have found that levels of PAI-1 and tPA were increased in patients with VTE (Bombeli et al., 2002). Patients with DVT were found to have lower mean fibrinolytic response to venous occlusion, higher antigen levels of t-PA and PAI-1. After venous occlusion, healthy controls had excess tPA over PAI-1, whereas only a minority of patients with VTE maintained that ratio (Grimaudo et al., 1992). Normal PAI-1 indicated normal fibrinolytic response in 97% of patients with idiopathic VTE, and can be used to detect 83% of patients with elevated PAI-1 (Grimaudo et al., 1992). Inability of t-PA released during venous occlusion to overcome inhibitory capacity of PAI-1 has been proposed as a biomarker that can predict risk of VTE, however it requires the use of the time-consuming venous occlusion test (Grimaudo et al., 1992).

Other coagulation factors were also discussed as possible biomarkers. Plasma levels of FVIII and vWF have been found to be elevated in patients with a history of at least one unprovoked VTE event (Bombeli et al., 2002), while the respective levels of vWF and factor VIII are well correlated in these patients and healthy controls (Bombeli et al., 2002). However, vWF levels did not correlate well with other endothelial derived proteins like soluble thrombomodulin (sTM), PAI-1, and tPA (Bombeli et al., 2002), which indicates differing secretion patterns.

#### Venous Thromboembolism Risk and Inflammation

Further complicating the picture is the role of inflammation. PAI-I, t-PA, and thrombin activatable fibrinolysis inhibitor (TAFI) are all associated with VTE, but so is plasminogen. The association of plasminogen with VTE seems to be surprising, since it is a precursor for the anti-fibrinolytic cascade, but plasminogen and t-PA levels may reflect underlying risk factors such as inflammation and endothelial activation (Masoud et al., 2008; Meltzer et al., 2010). This is supported by the findings that adjustment for acute phase protein attenuated the association between VTE and plasminogen, suggesting that plasminogen is a marker of systemic inflammation (Masoud et al., 2008).

The linkage between inflammation and VTE risk and clinical biomarkers does not always hold true. Reduced concentration of TFPI is a risk factor for development of DVT (Sidelmann et al., 2008). However, concentration of free and total TFPI, CRP, vWF and D-dimer were significantly higher in patients with clinically suspected VTE in an acute setting, but the relationship between these markers were not significantly linked with inflammation or endothelial cell disruption. It is believed TFPI may serve as a marker of endothelial cell dysfunction because inflammation and tissue injury are associated with elevated concentrations of TFPI, but the covariation between D-dimer and TFPI found by the above study suggests that D-dimer and TFPI are released from fibrin by degradation of fibrin clot. The TFPI relationship with acute DVT persisted when adjusted for effects of inflammation, which further supports that TFPI and inflammation are unlikely to be related (Sidelmann et al., 2008).

More direct indicators of inflammation, such as C-reactive protein (CRP), interleukin-6 (IL-6), and interleukin-8 (IL-8) are increased in patients with acute DVT (Bucek et al., 2002). Along the same line, high levels of markers of endothelial dysfunction such as vWF, P-selectin, and vascular cell adhesion protein (VCAM-1) are found in DVT patients (Jezovnik and Poredos, 2010). Van Aken et al. (Van Aken et al., 2000) found that the thrombosis risk for humans with IL-8 in upper quartile compared to lowest quartile was 9.4 (Van Aken et al., 2000). Interrelationship between inflammatory markers and markers of endothelial dysfunction favors the idea that inflammation could be involved in etiopathogenesis of idiopathic venous thrombosis. Another study found that patients with idiopathic VT had high levels of IL-6 and IL-8, as well as lower levels of the anti-inflammatory interleukin-10 (IL-10) during the stable phase of their disease (Poredos and Jezovnik, 2011). However, previous studies indicated that CRP levels do not seem to correlate with increased or decreased risk of VTE (Falkon et al., 1992), while others found that risk of VTE decreased after adjustment for markers of inflammation (Meltzer et al., 2010).

Chronic low-grade inflammation may be a risk factor for development of VTE as well as atherosclerotic disease, through endothelial dysfunction in the vasculature (Jezovnik and Poredos, 2010). The inflammatory process might not just be through alteration of procoagulant and antifibrinolytic activity but actual compromised endothelial integrity of the vessel wall that could be present in the stable phase of the disease as well. Inflammation could therefore be the inciting endothelial injury (Gordon and Lombard, 2017), or it could be a consequence of the disease, not a cause. There is a possibility that the coagulant process or thrombus itself induces inflammatory responses (Van Aken et al., 2000). Endothelial cell (EC) activation is associated with endothelial microparticle (EMP) release, and it has been found that patients with VTE had higher levels of EMP-62E (Chirinos et al., 2005). Elevated P-selectin, which mediates platelet endothelial interaction, shows that damage to the venous wall could be the inciting event (Jezovnik and Poredos, 2010).However, other studies found no difference concerning concentration of four major soluble cell adhesion molecules (sCAMS) between patients with DVT and controls in the acute phase (Bucek et al., 2003), which would indicate activation of vascular endothelial cells does not play a central role, at least acutely (Bucek et al., 2003).

Most studies look at these markers after patients have presented with their first VTE event, which does not provide any information about their premorbid state. Too much inflammation could reduce bioavailability of nitric oxide (NO), also reducing functional capacity of the vessel (Poredos and Jezovnik, 2011). If endothelial cell (EC) dysfunction is present, the antithrombotic potential of EC falls (Alvarado-Moreno et al., 2016). Activation of ECs due to cytokines may induce production of free radicals that allow ECs to malfunction (Alvarado-Moreno et al., 2016).

### Relevant Imaging and Diagnostic Techniques

All imaging described in the included studies focused on diagnosis of VTE, as opposed to establishing risk for VTE. Compression venous ultrasound (CUS) is considered the gold standard imaging for DVT (Bombeli et al., 2002; Bucek et al., 2002; Chirinos et al., 2005). CUS imaging is based on identifying deep venous incompressibility and thrombus visualization. The only imaging focusing on endothelial clotting risk was ultrasound measurement of flow mediated dilatation (FMD) of the brachial artery (Poredos and Jezovnik, 2011). FMD is a marker of pre-clinical arterial vessel wall remodeling processes and endothelial dysfunction (Raitakari and Celermajer, 2000). FMD has not been studied in the venous endothelial system.

### Identified Biomarkers

Several possible identifiers of biomarkers which are increased or decreased in the context of VTE were identified throughout this review. A summary in shown in Table 4. As highlighted in Table 2 above, most studies focused on the stable phase of VTE after the acute event had already occurred, therefore most of the below biomarkers are not predictive but rather reactive.

**TABLE 4:** Levels of various biomarkers in VTE patients versus healthy controls.

Parameter	Levels in VTE Patients Compared to Controls	Source
activation of platelets	Increased	Chirinos et al. (2005)
brachial artery FMD dilation	Decreased	Poredos et al. (2011)
Calprotectin	Increased	Alvarado-Moreno et al. (2016)
cfDNA	Increased	Alvarado-Moreno et al. (2016)
circulating APC	Decreased	Alvarado-Moreno et al. (2016)
CRP	Increased	Sidelmann et al. (2008), Jezovnik et al. (2010)
D-Dimer	Increased	Sidelmann et al. (2008)
E-selectin	Decreased	Torres et al. (2017)
ECFC	Increased	Alvarado-Moreno et al. (2016)
EMP	Increased	Chirinos et al. (2005)
Factor VIII	Increased	Torres et al. (2017)
	Increased^b^	Bombeli et al. (2002)
Fibrinogen	Increased	Torres et al. (2017)
formation of platelet-leukocyte conjugates	Increased	Chirinos et al. (2005)
hematocrit	Increased	Torres et al. (2017)
hemoglobin	Increased	Torres et al. (2017)
IFN-gamma	Increased	Alvarado-Moreno et al. (2016)
IL-10	Decreased	Poredos et al. (2011)
IL-6	Increased	Poredos et al. (2011), Jezovnik et al. (2010), van Aken et al. (2000), Alvarado-Moreno et al. (2016)
IL-8	Increased	Poredos et al. (2011), van Aken et al. (2000)
leukocytes	Decreased	Alvarado-Moreno et al. (2016)
lymphocytes	Decreased	Alvarado-Moreno et al. (2016)
MCP-1	Increased	van Aken et al. (2000)
myeloperoxidase	Increased	Alvarado-Moreno et al. (2016)
neutrophils	Decreased	Alvarado-Moreno et al. (2016)
P-selectin	Increased	Jezovnik et al. (2010)
PAI-1	Increased^a^	Meltzer et al. (2010)
	Increased^b^	Bombeli et al. (2002)
PAI-1 Ag levels	Increased^c^	Grimaudo et al. (1992)
plasminogen	Increased^a^	Meltzer et al. (2010)
Platelets	Decreased	Alvarado-Moreno et al. (2016)
TAFI levels	Increased^a^	Meltzer et al. (2010)
TFPI	Increased	Sidelmann et al. (2008)
TNF-alpha	Increased	Alvarado-Moreno et al. (2016)
tPA	Increased^a^	Meltzer et al. (2010)
	Increased^b^	Bombeli et al. (2002)
VCAM-1	Increased	Torres et al. (2017)
VEGF	Decreased	Alvarado-Moreno et al. (2016)
vWF	Increased	Sidelmann et al. (2008), Jezovnik et al. (2010), Torres et al. (2017)
	Increased^b^	Bombeli et al. (2002)

a higher risk of thrombosis.

b in patients with thrombosis.

c in patients with impaired fibrinolytic response to venous occlusion.

### Challenges of Studying Venous Endothelial Dysfunction

Fibrinolytic investigations are not commonplace because: 1) it is unclear what to look for to qualify as a fibrinolytic deficiency, 2) simulation tests are time consuming, and 3) there is no consensus on abnormal values (Grimaudo et al., 1992).

The study of venous endothelial dysfunction *in vivo* is not standardized; hence the proxy measurement of arterial dysfunction is often used instead (Poredos and Jezovnik, 2011). Endothelial dysfunction may not be restricted to the arterial system (Rühl et al., 2014), however dedicated study of venous endothelial dysfunction is not currently carried out even when investigating venous pathologies such as VTE.

The present review focused on biomarkers reflecting disturbance, activation, or inflammation of the venous wall. Unfortunately, due to the nature of the measurement methods used in the studies, the venous system was not particularly distinct from the arterial one or from systemic markers of inflammation, such as CRP.

### Status of the Current Evidence Base

The lack of longitudinal studies related specifically to the biomarkers outlined above is another weakness of the included studies. Most published studies focus either on the immediate acute biomarkers present in patients presenting with VTE, or in the chronic phase of the disease, as seen in Table 2. The proceeding state of the patients, aside from the exclusion of patients based on known risk factors such as hormonal birth control, thrombophilia, and atherosclerotic disease, was unknown. The small number of studies in this area limited our analysis of each specific biomarker and its broader clinical context.

The causal nature of venous wall inflammation and VTE is yet to be elucidated (Van Aken et al., 2000). More studies are required to explore the link between neutrophil adhesiveness and VTE recurrence (Zapponi et al., 2011). Further studies should also be dedicated to variation in fibrinolysis with time of day (Ansari et al., 2006).

Further predictive value for each of the biomarkers outlined in Table 4 above for long term risk as well as acute incidence of VTE needs to be assessed. A prospective study focusing on each of the above biomarkers would further clarify their role as diagnostic and/or predictive markers of VTE, and contribute to the understanding of the etiology of VTE and the role of the various factors.

With a greater understanding of the roles that possible predictive biomarkers may play in the pathogenesis of VTE, targeted therapies may be developed significantly reducing the risk of VTE (Martos et al., 2020).

## Conclusion

This review sought to identify biomarkers linked to venous endothelial dysfunction on Earth that can be used to elucidate mechanisms of spaceflight-associated VTE. The analysis of the 17 included papers found that distinction between the arterial and venous systems, as well as between local and systemic inflammation was lacking. Routine imaging to identify risk of VTE was not well studied. Most available studies examined biomarkers after the acute event, which provided limited predictive value for each of the biomarkers identified.

Future work should focus on conducting prospective cohort studies to identify levels of specific endothelial biomarkers that can accurately assess risk of VTE to enable better patient management before exposure to known or potential triggers of thromboembolic events, such as spaceflight. Future studies should additionally focus on the differences between the arterial and venous systems to further understand the pathogenesis of VTE.

## Data Availability

The original contributions presented in the study are included in the article/Supplementary Material, further inquiries can be directed to the corresponding author.

## Abbreviations

CRP, C-reactive protein; CUS, Compression venous ultrasound; DVT, Deep vein thrombosis; EC, Endothelial cell; EMP, Endothelial microparticle; ESA, European Space Agency; FMD, Flow mediated dilatation; FVa, Activated Factor V, FVIIIa; Activated Factor VIII; IL-10, Interleukin 10; IL-6, Interleukin-6; IL-8, Interleukin-8; MeSH, Medical subject headings; PAI-1, Plasminogen activator inhibitor 1; PAP, Plasmin-alpha2-antiplasmin; PC, Protein C; PICO, Population, Interest, Control, and Outcome; PS, Protein S; sCAMS, Soluble cell adhesion molecule; sTM, Soluble thrombomodulin; TAFI, Thrombin activatable fibrinolysis inhibitor; TF, Tissue factor; TFPI, Tissue factor plasminogen inhibitor; tPA, Tissue plasminogen activator; VCAM-1, Vascular cell adhesion protein 1; VTE, Venous thromboembolism; vWF, Von Willebrand factor.
