# Supplementary material for: Search for Venous Endothelial Biomarkers Heralding Venous Thromboembolism in Space: A Qualitative Systematic Review of Terrestrial Studies
Source: Front Physiol. 2022 Apr 27;13:885183. doi: 10.3389/fphys.2022.885183 (PMC9092216; doi:10.3389/fphys.2022.885183)
Supplement: Supplementary file 1 [file Table1.DOCX]

Supplementary Material

Supplementary Table 1: Population, Interest, Control, Outcome Table with search strategy.

| **Category** | **Specific Category** | **Keywords** | **Search Number** | **Search Mask** |
| --- | --- | --- | --- | --- |
| Population | N/A | “human*” OR “homo sapien*” OR “women” OR “woman” OR “man” OR “men” OR “female” OR “male” OR “adult*” OR “Humans” [MeSH] | 1 | All fields |
| Interest | Venous thromboembolism | “venous thrombo*”[tiab] OR “VT” [tiab] OR “VTE” [tiab] OR “clot”[tiab] OR “embol*”[tiab] OR “DVT”[tiab] | 2 | Title/abstract |
|  | Endothelial function | “endothel*”[tiab] OR “intima”[tiab] OR “EC”[tiab] | 3 | Title/abstract |
|  | Exclusions | “cancer”[tiab] OR “tumor*”[tiab] OR “malignanc*”[tiab] OR “neoplasm*”[tiab] OR “COVID-19” [tiab] OR “SARS CoV 2” [tiab] OR “Coronavirus”[tiab] OR “pregnan*”[tiab] | 4 | Title/abstract |
| Control | N/A | N/A |  | N/A |
| Outcome | Structural Biomarkers | “structural biomark*” OR “vessel wall thickness” OR “wall structural change*” OR “venous thrombogenesis” OR  “cranial ve*” OR “Biomarkers”[MeSH] OR “Cerebral Blood Volume”[MeSH] | 5 | All fields |
|  | Venous mechanical properties | “mechanical propert*” OR “venous compliance” OR “wall extensibility” OR “elastic modulus” OR “veins”[MeSH] OR “elastic modulus”[MeSH] OR “Mechanical Phenomena”[MeSH] OR “vasoconstriction”[MeSH] OR “vasodilation”[MeSH] | 6 | All fields |
|  | Venous flow properties | “venous flow” OR “venous flow direction” OR “venous flow velocity” OR “venous flow volume” OR “Venous blood pressure” OR “venous BP” OR “venous pressure”[MeSH] | 7 | All fields |
|  | Symptoms | “symptom*” OR “pain” OR “swelling” OR “edema” OR “diagnosis”[MeSH] OR “pain”[MesH] OR “edema”[MeSH] | 8 | All fields |
|  | Circulating Biomarkers | “circulating biomarker*” OR “Soluble P-SELECTIN” OR  “inflammatory cytokine*” OR  “ICAM-1” OR “intercellular adhesion molecule-1” OR “cell free DNA” OR “interleukin-6” OR “IL-6” OR “IL-8” OR “interleukin-8” OR “IL-10” OR “interleukin-10” OR “p-selectin”[MeSH] OR “intercellular adhesion molecule-1”[MeSH] OR “cell-free nucleic acids” [MeSH] OR “interleukin-6”[MeSH] OR “interleukin-8”[MeSH] OR “interleukin-10”[MeSH] | 9 | All fields |
|  | Endothelial markers | “endothelial marker*” OR “Tissue Factor” OR “TF” OR  “Tissue-plasminogen activator” OR “tPA” OR “thromboplastin”[MeSH] OR “tissue plasminogen activator”[MeSH] | 10 | All fields |
|  | Blood cell counts | “blood cell count*” OR “complete blood count” OR “CBC” or “white blood cell*” OR “WBC” OR “red blood cell*” OR “RBC” or “hemoglobin” OR “haemoglobin” OR “Hb” OR “hematocrit” or “haematocrit” OR “Hct” OR “platelet*” OR “blood cell count”[MeSH] OR “hemoglobins”[MeSH] OR “hematocrit”[MeSH] | 11 | All fields |
|  | Thrombelastometry | “thrombelastomet*” OR “TEM” OR “EXTEM” OR “INTEM” OR “FIBTEM” OR “thromboelastograph*” OR “TEG” OR “Sonoclot” OR “CT” OR “clotting time” OR   “CFT” OR “clot formation time” OR “MCF” OR “maximum clot firmness” OR “fibrin clot lysis time”[MeSH] | 12 | All fields |
|  | Platelet Aggregation | “platelet aggreg*” OR “platelet aggregation”[MeSH] | 13 | All fields |
|  | Platelet adhesion | “platelet adhes*” OR “surface cover*” OR “platelet adhesiveness” [MeSH] | 14 | All fields |
|  | Coagulation times | “coagulation” OR “APTT” OR “activated partial thromboplastin clotting time” OR “partial thromboplastin time” OR “PTT” OR “INR” OR “prothrombin” OR “prothrombin time” OR “PT” “thrombin time” OR “activated whole blood clotting time” OR “ACT” OR “anti-factor Xa” OR “anti-Xa” OR “D-dimer” OR “international normalized ratio” OR “blood coagulation”[MeSH] OR “blood coagulation tests”[MeSH] | 15 | All fields |
|  | Thrombin generation | “thrombin generat*” OR “prothrombin fragment” OR “F1+2” OR “TAT” OR “thrombin antithrombin complex” OR “protein C system” OR “thrombomodulin” OR “lag time” OR “ETP” OR “endogenous thrombin potential” OR “peak” OR “time to peak” OR “start tail” OR “prothrombin fragment 1.2” OR “antithrombin III-protease complex” OR “Protein C”[MeSH] OR “Thrombomodulin”[MeSH] | 16 | All fields |
|  | Fibrinolytic values/endothelial activation | “fibrinolytic parameter*” OR “endothelial activ*” OR “t-PA Ag” OR “tissue plasminogen activator” OR “tPA” OR “plasminogen activator inhibitor 1” OR “PAI-1 Ag” OR “TF” OR “tissue factor” OR “EndoPAT” OR “RHI” OR “reactive hyperemia index” OR “ADMA” OR “Asymmetric dimethylarginine  “ OR “pulse wave velocity” OR “nitric oxide” OR “NO” OR “microvasculature” OR “retinal imag*” OR “Plasminogen Activator Inhibitor 1”[MeSH] | 17 | All fields |
|  | Procoagulatory factors | “procoagulation factor*” OR “F II” OR “factor II” OR “F VII” OR “factor VII” OR “F VIII” OR “factor VIII” OR “VWF” OR “von Willebrand factor” OR “Fibrinogen” OR “microparticle*” OR “Blood Coagulation Factors”[MeSH] | 18 | All fields |
|  | Anticoagulatory factor | “anticoagulation factor*” OR “Protein C” OR “protein S” OR “antithrombin” OR “TFPI” OR “tissue factor pathway inhibitor” OR “Blood Coagulation Factor Inhibitors”[MeSH] | 19 | All fields |
|  |  | #5 OR #6 OR #7 OR #8 OR #9 OR #10 OR #11 OR #12 OR #13 OR #14 OR #15 OR #16 OR #17 OR #18 OR #19 | 20 |  |
|  |  | #1 AND #2 AND #3 NOT #4 AND #20 | 21 |  |
